# Heterogeneity of *Moraxella* isolates found in the nasal cavities of piglets

**DOI:** 10.1186/s12917-020-2250-9

**Published:** 2020-01-30

**Authors:** Sergi López-Serrano, Nuria Galofré-Milà, Mar Costa-Hurtado, Ana M. Pérez-de-Rozas, Virginia Aragon

**Affiliations:** 1 IRTA, Centre de Recerca en Sanitat Animal (CReSA, IRTA-UAB), Campus de la Universitat Autònoma de Barcelona, 08193 Bellaterra, Spain; 2 OIE Collaborating Centre for the Research and Control of Emerging and Re-emerging Swine Diseases in Europe (IRTA-CReSA), Bellaterra, Barcelona, Spain

**Keywords:** Swine, Nasal microbiota, Virulence, Bacterial diversity, *Moraxella*

## Abstract

**Background:**

Previous studies have shown that the genus *Moraxella* is commonly present in the nasal microbiota of swine.

**Results:**

In this study, 51 isolates of *Moraxella* were obtained from nasal swabs from 3 to 4 week old piglets, which represented 26 different fingerprintings by enterobacterial repetitive intergenic consensus (ERIC)-PCR. Whole 16S rRNA gene sequencing allowed the identification at species level of the *Moraxella* spp. isolates. The majority of the field strains were identified as *Moraxella pluranimalium,* but *Moraxella porci* was also detected. In addition, a cluster of 7 strains did not group with any described *Moraxella* species, probably representing a new species. Subsequent phenotypic characterization indicated that strains of *Moraxella pluranimalium* were mainly sensitive to serum complement, while the cluster representing the putative new species was highly resistant. Biofilm formation capacity was very variable among the *Moraxella* spp. isolates, while adherence to epithelial cell lines was similar among selected strains. Additionally, variability was also observed in the association of selected strains to porcine alveolar macrophages. Antimicrobial tests evidenced the existence of multidrug-resistance in the strains.

**Conclusions:**

In summary, phenotypic characterization revealed heterogeneity among *Moraxella* strains from the nasal cavity of piglets. Strains with pathogenic potential were detected as well as those that may be commensal members of the nasal microbiota. However, the role of *Moraxella* in porcine diseases and health should be further evaluated.

## Background

*Moraxella* is a microbiota member of the upper respiratory tract in vertebrates, but some species may cause opportunistic infections. Best-known *Moraxella* infections are produced by *Moraxella catarrhalis*, which causes respiratory infections, including pneumonia and otitis in human [1]. In livestock, *Moraxella bovis* is well-known as the etiological agent of infectious keratoconjunctivitis in cattle [2]. In swine, the genus *Moraxella* has representative species, such as *Moraxella porci*, which was discovered and isolated from meninges [3] and *Moraxella pluranimalium*, described from an abdominal cavity isolate [4], but the relevance of this genus in swine health is not well established. Nonetheless, *Moraxella* may play a role in swine as a member of the nasal microbiota, since it has been detected as one of the most abundant genera in the nasal cavity of weaned piglets [5], is abundant in the nasal microbiota of slaughter age pigs [6] and can be found in environmental samples in farrowing buildings [7].

The microbiota plays an important role in host health and disease through different mechanisms, such as maturation of the immune system, improvement of the mucosal barrier and resistance against pathogens [8]. In the case of the nasal microbiota, early colonizers can determine the stability and composition of bacterial community leading to a healthier status, as reported for children early colonized by high abundance of *Moraxella*, which was associated with fewer respiratory infections [9]. However, the specific role of *Moraxella,* among other residents of the respiratory microbiota, in health is still unknown in pigs. In this study, we performed a genotypic and phenotypic characterization of 51 *Moraxella* field isolates from the nasal turbinates of weaned piglets. We assessed their capacity of evasion of the innate immunity by examining their complement susceptibility and macrophage association capacity, as well as other characteristics closely related to its ecological niche, such as biofilm formation, mucin adhesion and cell adherence. Our results showed genotypic and phenotypic heterogeneity among the *Moraxella* isolates.

## Results

### Bacterial identification and genotyping

**Fig. 1:**
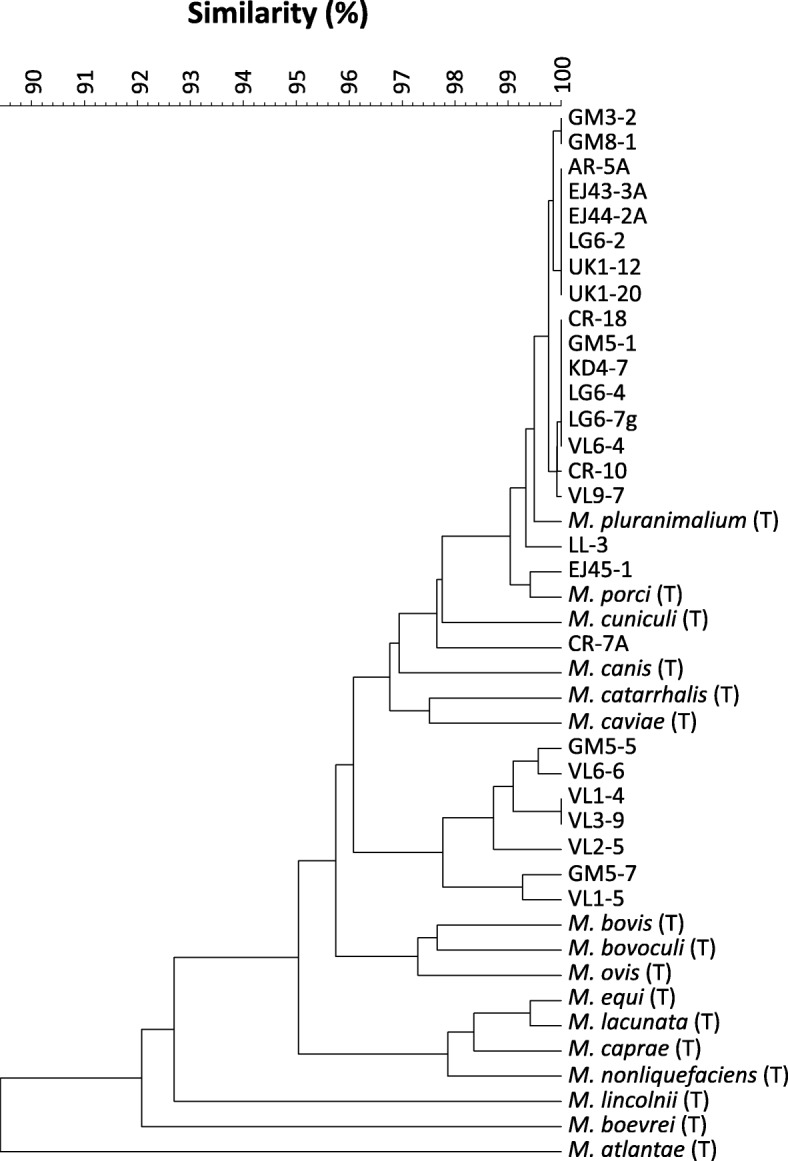
UPGMA tree built with the 16S rRNA gene sequences of the *Moraxella* spp. isolates from the nasal cavities of piglets at weaning. Type strains (T) of different *Moraxella* species were included in the analysis

Initial identification of bacterial isolates by partial 16S rRNA gene sequencing showed a total collection of 51 *Moraxella* spp. isolates. Genotyping by enterobacterial repetitive intergenic consensus (ERIC)-PCR determined 26 different ERIC fingerprintings, which were not shared among farms (Table 1). Up to 7 different fingerprinting profiles were isolated from the same farm, and up to 3 from a single piglet. One isolate of each fingerprinting (from now on referred as different strains for clarity throughout the text) were selected for further analysis. Sequencing of approximately 1360 bp of the 16S rRNA gene allowed a more precise identification of the *Moraxella* isolates. Sequences from the nasal isolates and sequences from *Moraxella* sp. type strains from the Ribosomal database were used to build an UPGMA (unweighted pair group method with arithmetic mean) tree (Fig. 1). Sixteen nasal strains clustered with more than 99.5% identity with *Moraxella pluranimalium*, while one strain, LL-3, was a bit more divergent, with 99.4% identity. Strain EJ45–1 clustered with *Moraxella porci*, with 99.2% identity. Strain CR-7A showed similarity to *M. porci* and *Moraxella cuniculi,* but did not clearly cluster with any of them*.* Finally, a group of 7 nasal strains showed homology among them of more than 98%, but did not clustered with any of the already described *Moraxella* species, indicating that they may represent a new species. Biochemical characterization of representative isolates can be found in Additional file 1.

**Table 1:** Sampled farms and nasal *Moraxella* spp. isolated from piglets at weaning (3–4 weeks of age). Ten piglets representing 5 litters from each farm were sampled. Name of strain indicates farm of origin, followed by piglet number and finally the number of isolate. Exceptions to this are LL and CR isolates, which indicate only the number of isolate

Farm	Selected strains	Isolates with same ERIC PCR profile	Health status	Antimicrobial treatment^a^
GM	GM3–2	GM4–1	Healthy	AMOX
	GM8–1	–		
	GM5–1	GM7–2, GM9–1, GM10–1		
	GM5–5	GM7–6		
	GM5–7	GM7–8, GM7–9, GM8–4		
AR	AR-5A	AR-5C	Healthy	AMOX
EJ	EJ43-3A	–	Glässer’s disease	ENR
	EJ44-2A	–		
	EJ45–1	EJ45–5		
UK/LG	UK1–12	UK1–32, LG1–4, LG1–6, LG2–2, LG3–7	Healthy	–
	UK1–20	LG5–5, LG8–3		
	LG6–2	–		
	LG6–4	LG6–6		
	LG6-7 g	–		
CR	CR-18	–	Healthy	AMOX
	CR-10	–		
	CR-7A	CR-12		
KD	KD4–7	–	Healthy	–
VL	VL6–4	–	Healthy	TUL, CEFT
	VL9–7	VL9–8, VL10–1, VL10–2		
	VL6–6	–		
	VL1–4	VL1–7, VL5–3		
	VL3–9	–		
	VL2–5	–		
	VL1–5	VL1–6		
LL	LL-3	–	Nervous signs	Unknown

*AMOX* Amoxicillin, *ENR* enrofloxacin, *CEFT* ceftiofur, *TUL* tulathromycin

^a^Antibiotics administered before weaning (before sampling)

### Antimicrobial susceptibility

*Moraxella* strains from nasal microbiota showed high diversity in antibiotic susceptibility (Table 2). In general, all the strains were sensitive to amoxicillin+clavulanate and florfenicol, in contrast to trimetoprim + sulfonamide and tetracycline, where we found a high resistance rate. Other antimicrobials with low resistance rates were ceftiofur, gentamicin and colistin. Six strains showed resistance to amoxicillin, which was abolished with the β-lactamase inhibitor clavulanate. *M. pluranimalium* UK1–12 and UK1–20 showed only resistance to tetracycline; both strains were isolated from the same farm, which did not use antimicrobials before the sampling time. Six strains were resistant to 2 different antimicrobials. Multidrug-resistant (MDR), defined as resistance to 3 or more classes of antimicrobials, was found in 18 out of the 26 strains. A higher percentage of MDR strains was observed in the farms using antimicrobials than in those not using these drugs during lactation (75% versus 50%, respectively), although no statistical significance was found.

**Table 2:** Antimicrobial susceptibility of nasal *Moraxella* strains isolated from 3 to 4 week old piglets

Strain	COL	ENR	TET	DOX	S + T	AMOX	AMC	CEFT	GEN	LI + SP	ERY	TUL	MARB	FLOR
*M. pluranimalium*														
GM3–2	S (25.6)	I (18.7)	R (9)	S (27.3)	R (9)	S (20.4)	S (38.5)	S (28.2)	S (21.3)	S (21.2)	S (28.7)	S (0.5)	R (8)	S (1)
GM8–1	S (24.4)	I (17.6)	R (9)	S (24.8)	I (15.8)	I (17.6)	S (36.4)	S (28.3)	S (23.9)	S (20)	S (24.5)	S (0.5)	R (8)	S (1)
AR-5A	S (26.5)	I (20.9)	R (9)	I (20.7)	R (9)	R (15.9)	S (38.2)	S (23.9)	S (24)	R (14.5)	S (28.2)	S (16)	R (4)	S (1)
EJ43-3A	S (28)	I (19.6)	I (20.3)	S (24.2)	S (27)	R (13.4)	S (33.1)	I (18.7)	S (22.9)	R (9)	R (9)	R (> 64)	R (8)	S (2)
EJ44-2A	R (9)	R (16.1)	R (9)	R (19.7)	R (9)	R (9)	S (29.9)	R (14.5)	R (9)	R (9)	S (24.5)	S (16)	R (4)	S (1)
LG6–2	S (21)	S (35.9)	R (13.6)	I (20.8)	R (9)	I (19.6)	S (33)	S (30.7)	S (17.5)	S (20.6)	S (25.4)	S (4)	S (0.125)	I (4)
UK1–12	S (25.8)	S (42.6)	R (13.2)	S (26)	S (17.5)	S (21)	S (39.5)	S (27.2)	S (23.9)	S (20.3)	S (28.7)	S (1)	S (0.0625)	S (0.5)
UK1–20	S (33.4)	S (41.5)	R (18.5)	S (32.8)	S (41.1)	S (23.5)	S (38.4)	S (30.3)	S (27.5)	S (29.6)	S (31.9)	S (2)	S (0.0625)	S (0.5)
CR-18	S (27.7)	S (26.5)	R (9)	S (29.2)	R (9)	R (13.2)	S (36.2)	S (26.1)	S (21.5)	S (28.9)	S (26.1)	S (0.5)	S (0.5)	S (0.5)
GM5–1	S (23.6)	I (22.6)	R (9)	S (25.8)	I (15.7)	S (22.7)	S (37.7)	S (27.6)	S (25.1)	S (30.7)	S (28.8)	S (16)	R (4)	S (0.5)
KD4–7	S (25.2)	S (28.2)	R (15.7)	S (24.9)	R (9)	S (33.9)	S (45.7)	S (33.4)	S (25.3)	S (31.6)	S (30)	S (2)	R (16)	S (0.5)
LG6–4	S (23.1)	S (30.4)	R (11.9)	I (23.6)	R (9)	I (16.5)	S (36.4)	S (29.9)	I (15.4)	R (15.1)	S (25.9)	S (8)	S (1)	S (1)
LG6-7 g	S (23)	S (28)	R (12.5)	I (22.4)	R (9)	I (19.7)	S (37.5)	S (31.6)	I (15.4)	R (13.6)	S (28.3)	S (8)	R (8)	I (4)
VL6–4	R (9)	I (18.1)	R (9)	I (20.7)	R (9)	R (9)	S (29.9)	S (23.3)	R (9)	R (9)	R (9)	R (> 64)	R (4)	S (2)
CR-10	S (31.6)	I (18.4)	R (9)	I (23.6)	R (9)	I (17.9)	S (38.9)	S (23.4)	S (21.8)	I (19.2)	S (30)	S (0.5)	R (8)	S (1)
VL9–7	I (18.3)	I (21.4)	R (9)	S (29.3)	R (9)	S (20.8)	S (39.4)	S (26.7)	S (26.8)	R (9)	R (9)	R (> 64)	R (4)	S (1)
LL-3	S (20.5)	I (17.1)	R (9)	I (23.3)	R (9)	I (17.4)	S (34.9)	I (21.1)	S (18.6)	I (16.6)	I (20.6)	S (8)	S (1)	S (0.5)
*M. porci*														
EJ45–1	R (14.5)	I (19.7)	R (9)	R (9)	S (22.1)	S (23.3)	S (40.6)	S (33.8)	R (9)	S (37)	S (29.5)	S (4)	I (2)	S (0.125)
Other *Moraxella*														
GM5–5	S (27.5)	S (27)	R (9)	S (34.2)	R (9)	I (19.2)	S (41.4)	S (33.7)	R (9)	S (24)	R (9)	R (64)	S (1)	S (0.5)
VL6–6	S (29.2)	S (31.7)	R (9)	S (33.7)	R (9)	S (41.3)	S (57.3)	S (51.2)	S (29.8)	S (65.6)	R (9)	R (> 64)	S (1)	S (0.5)
VL1–4	R (9)	I (20.5)	R (9)	I (21.8)	R (9)	S (29.1)	S (52.9)	S (33.4)	S (40.1)	S (23.8)	R (9)	R (> 64)	R (4)	S (2)
VL3–9	S (18.7)	I (20.5)	R (9)	I (22.6)	R (9)	R (12.5)	S (55.5)	S (41.1)	S (32.7)	S (35.6)	R (9)	R (> 64)	I (2)	S (2)
VL2–5	S (34.4)	S (42.3)	R (19.6)	S (38.5)	R (9)	S (29.8)	S (52.5)	S (40.3)	S (27.8)	I (18.3)	R (9)	S (0.125)	S (0.0625)	S (0.125)
GM5–7	S (27.6)	S (> 30)	R (15.5)	S (36.9)	S (50.2)	S (61.4)	S (60)	S (62.6)	S (31.9)	S (22.6)	R (9)	R (64)	S (0.625)	S (0.125)
VL1–5	S (26.3)	S (42.7)	R (9)	S (33.2)	I (15.2)	S (65.4)	S (67.6)	S (56.4)	S (31.3)	S (59)	R (9)	S (< 0.125)	S (0.25)	S (0.125)
CR-7A	S (26.7)	I (17.8)	R (9)	S (24)	R (9)	S (20.8)	S (39.6)	S (28.3)	S (23.6)	I (19.2)	S (29.7)	S (0.5)	R (8)	S (1)

Diffusion disk breakpoints in mm: *COL* colistin S ≥ 20 R ≤ 16, *ENR* enrofloxacin S ≥ 23 R ≤ 16, *TET* tetracycline S ≥ 24 R ≤ 20, *DOX* doxycycline S ≥ 24 R ≤ 20; S + T, trimethroprim-sulfonamide S ≥ 16 R ≤ 10, *AMOX* amoxicillin S ≥ 20 R ≤ 16, *AMC* amoxicillin + clavulanate S ≥ 30 R ≤ 27, *CEFT* ceftiofur S ≥ 21 R ≤ 17, *GEN*, gentamicin S ≥ 16 R ≤ 12, *LI + SP* lincomycin-spectinomycin S ≥ 20 R ≤ 16, *ERY* erythromycin S ≥ 23 R ≤ 13

*MIC* breakpoints in μg/ml, *TUL* tulathromycin S ≤ 16 R ≥ 64, *MARB* marbofloxacin S ≤ 1 R ≥ 4, *FLOR* florfenicol S ≤ 2 R ≥ 8

### Serum resistance

Initially, bacterial suspensions were prepared in PBS obtaining irregular results, due to auto-agglutination by some strains. To overcome this problem, all the strains were resuspended in 20% glycerol in PBS and passed through a needle if necessary; this process did not affect the viability of the bacteria (not shown).

**Fig. 2:**
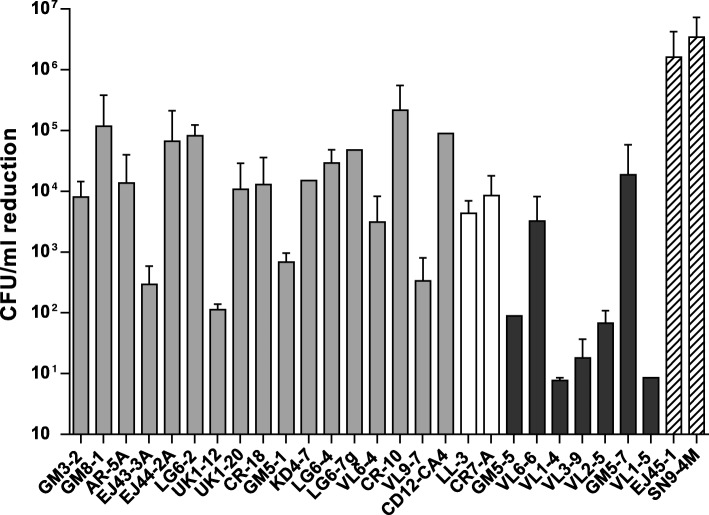
Serum resistance assay showed diverse CFU reduction among the nasal *Moraxella* spp. strains. Survival of *Moraxella pluranimalium* strains (gray bars), uncertainly classified *Moraxella* strains (white bars), strains from the putative new *Moraxella* species cluster (black bars) and *M. porci* (streaked bars) was tested with 90% rabbit serum. Initial bacterial inoculum was approximately 10^6^ CFU/ml. Results are expressed as average of the log CFU reduction after 1 h of incubation at 37 °C with the serum, error bars indicate standard deviation from three replicate assays

When the strains were assayed for survival after incubation with fresh rabbit serum, 17 of the 26 (65%) strains showed a reduction in viability of more than 3 logarithms after incubation with serum (Fig. 2). Within *M. pluranimalium*, 12 out of the 16 strains (75%) showed a reduction of viability of more than 3 logs, similar to the type strain of this species, while the rest 4 (25%) showed a reduction of 2–3 logs. On the other hand, only 2 of the 7 (29%) strains from the cluster representing a putative new *Moraxella* species showed a reduction of more than 3 logs in viability, while the rest of strains in this cluster (5/7; 71%) showed a reduction of viability of less than 2 logarithms (Fig. 2), indicating resistance to the serum complement. Moreover, strains showing the highest resistance to serum were observed within the isolates from the cluster of the putative new *Moraxella* species, and belonged to the same farm (VL1–4, VL3–9 and VL1–5; Fig. 2). *M. porci* type strain SN9-4 M, as well as strain *M. porci* EJ45–1, showed high susceptibility to the treatment with rabbit sera (Fig. 2).

### Biofilm assays

**Fig. 3:**
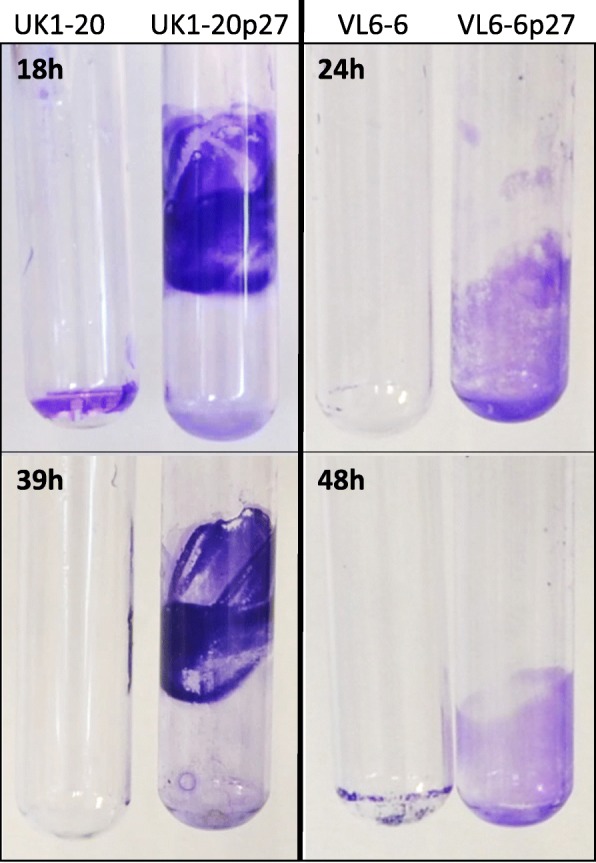
Effect of culture passages on biofilm formation. Biofilm produced in glass tubes by strains UK1–20 and VL6–6 after 27 culture passages in the laboratory (UK1–20p27 and VL6–6p27). The original strains are included in the pictures for comparison. Cultures were incubated for different times (indicated in the figure) and pictures were taken after elimination of the bacterial culture and staining with crystal violet. Although it is not clear in the picture, VL6–6p27 formed biofilm as a floating pellicle at 24 h

**Fig. 4:**
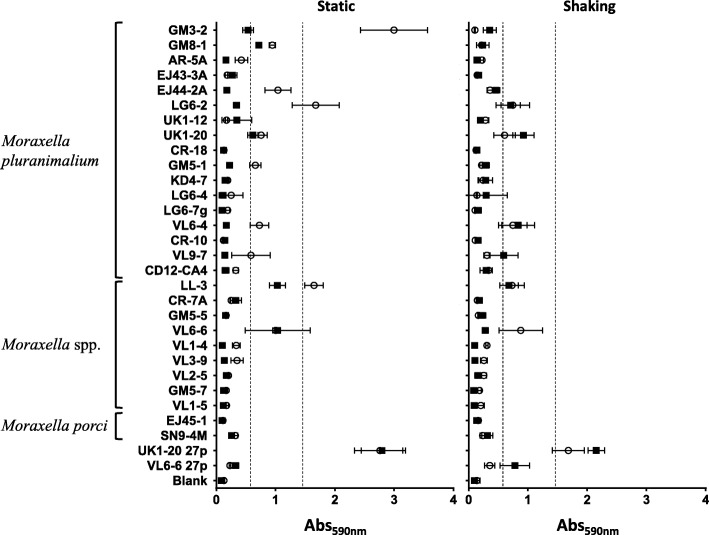
Biofilm formation by *Moraxella* spp. isolates. Biofilm formation under static and shaking conditions in microtiter plates for *Moraxella* spp. nasal strains. Biofilm was measured at 24 h (open circles) and 48 h (black squares) by staining with crystal violet. In slow growers (cluster of putative new *Moraxella* species GM5–5, GM5–7, VL1–4, VL1–5, VL2–5, VL3–9, VL6–6) incubations were 48 h and 72 h (open circles and black squares, respectively). Quantification of biofilm was determined the absorbance at 590 nm of the dissolved dye. Strains UK1–20 and VL6–6 after 27 in vitro culture passages (UK1–20p27 and VL6–6p27) were included in the assay. Blank wells with uninoculated medium were also processed and included in the figure. Vertical dashed lines indicate the levels used in this study to consider that a strain had ability to form biofilm (Abs590 ≥ 0.6) or strong biofilm (Abs590 > 1.5)

In an attempt to select non-agglutinating variants, we performed several passes of the agglutinating strains UK1–20 and VL6–6 in broth culture. Each passage was performed with bacteria taken from the surface of the culture that did not agglutinate. Curiously, a thick biofilm was observed in the culture tubes of UK1–20 after 27 passes (UK1–20p27); the bacteria in these cultures did not agglutinate but stuck to the glass. In the case of VL6–6, a biofilm in the air-liquid interphase was observed after 27 passes (VL6–6p27) (Fig. 3). This observation prompted us to examine the natural level of biofilm formation capacity of the rest of the nasal *Moraxella* sp. strains. We found different rates of biofilm formation that were not dependent on the *Moraxella* species. Observing the results, different levels of biofilm formation could be arbitrarily established: production of biofilm when Abs590 ≥ 0.6 and of strong biofilm when Abs_590_ > 1.5. With those definitions, strains showing biofilm production capacity were *M. pluranimalium* GM3–2, GM8–1, EJ44-2A, LG6–2,, UK1–20, GM5–1, VL6–4 and VL9–7; *Moraxella* sp. LL-3; *Moraxella* sp. (putative new species cluster) VL6–6; and also the variants UK1–20p27 and VL6–6p27 used as controls (Fig. 4). Few isolates (*M pluranimalium* GM3–2 and LG6–2, and *Moraxella* sp. LL-3) were able to form strong biofilms (Fig. 4). In general, strains forming biofilm under static conditions formed biofilm also under shaking conditions, although at different level. Under static conditions, we observed a common trend of decreasing biofilm production when comparing 24 and 48 h. In contrast, we observed the opposite effect under shaking conditions, with most of the strains showing a tendency of increasing biofilm production with time. Strains with slow growth (unclassified cluster) showed the same trend in decreasing biofilm production under both conditions. Biofilm formation was not affected by pre-coating the wells with mucin or bovine serum albumin (BSA) (data not shown).

### Cell adhesion

**Fig. 5:**
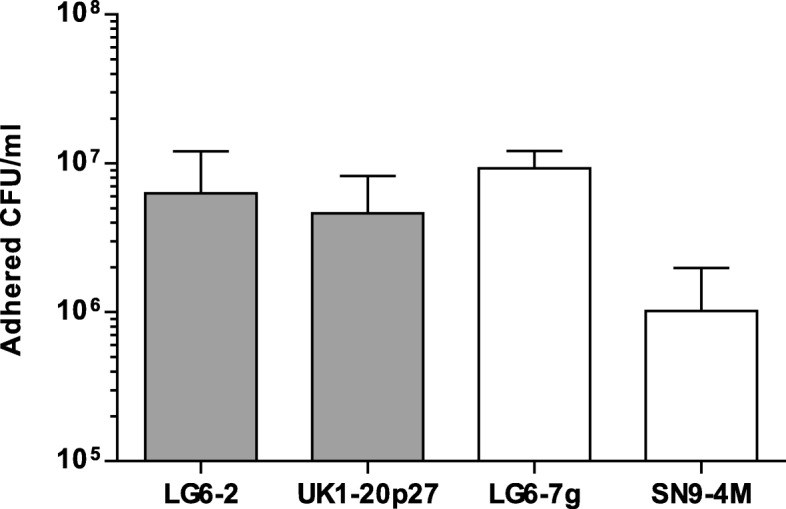
Bacteria adhesion to A549 cells. Two biofilm forming strains, LG6–2 and UK1–20p27, and two non-forming strains, LG6-7 g and SN9-4 M, were incubated with pulmonary A549 cells to examine their adhesion capacity. After 1 h of incubation, non-attached bacteria were washed and attached bacteria were quantify by dilutions and plating. Results represent the average adherent bacteria from 3 different assays (error bars: standard deviation)

To explore whether the biofilm formation was associated with cell adhesion capacity, selected strains with different rates of biofilm formation were assayed in PK-15 and A549 cell lines. Although the assayed strains presented adhesion ability to cells, the observed differences in biofilm formation capacity did not correlated with differences in cell adhesion to both PK-15 (not shown) or A549 cells (Fig. 5).

### Association to porcine alveolar macrophages

**Fig. 6:**
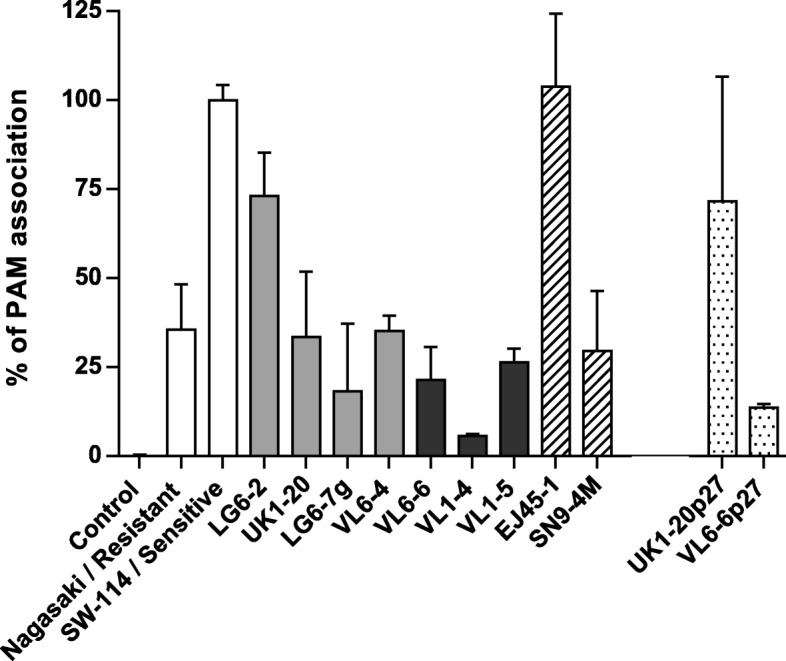
Phagocytosis susceptibility of selected *Moraxella* spp. strains. Selected *M. pluranimalium* strains (gray bars), strains from the putative new species cluster (black bars) and *M. porci* strains (streaked bars). FITC-labelled *Moraxella* spp. strains were incubated with porcine alveolar macrophages (PAMs) during 1 h at 37 °C. After washing to eliminate unbound bacteria, association of bacteria with PAMs was measured by flow cytometry and expressed as % of fluorescent PAMs. *H. parasuis* susceptible strain SW114 and resistant strain Nagasaki were included as control for the assay (white bars) and SW114 levels of phagocytosis were used to normalize the results of different tests. In addition, strains UK1–20 and VL6–6 after 27 passages on agar plates were also tested (dotted bars). Results represent the average of duplicate wells from 2 different assays (error bars: standard deviation)

Selected strains representing the diversity in phylogenetic, serum resistance and biofilm formation were tested with porcine alveolar macrophages (PAMs). Different levels of association with PAMs were observed among the strains (Fig. 6). Within the 4 *Moraxella pluranimalium* strains tested, only LG6–2 presented a clear association with PAMS, similar to the level observed in the susceptible *H. parasuis* strain used as control. *Moraxella porci* EJ45–1, unlike the *M. porci* type strain SN9-4 M, showed high levels of association with PAMs. The three isolates from the putative new species cluster presented low association with macrophages, even lower than the *H. parasuis* phagocytosis resistant strain used as control (Fig. 6). Levels of association to macrophages were not directly linked to biofilm formation capacity or serum resistance (Table 3).

**Table 3:** Summary of results from serum susceptibility, association to alveolar macrophages and biofilm formation capacity for selected *Moraxella* spp. Isolates

Strains	Biofilm formation^b^	Serum susceptibility^c^	Association to macrophages
*M. pluranimalium*			
LG6–2	+	S	S
UK1–20	+	S	R
LG6-7 g	–	S	R
VL6–4	+	S	R
*Moraxella* sp.^a^			
VL6–6	+	S	R
VL1–4	–	R	R
VL1–5	–	R	R
*M. porci*			
EJ45–1	–	S	S
SN9-4 M	–	S	R

^a^Strains in the putative new *Moraxella* species cluster

^b^Abs590 > 0.8 in the crystal violet assay

^c^Viability reduction of more than 3 logarithms

#### In vitro culture passages affected association to macrophages

In vitro passages affected the autoagglutination and biofilm formation capacity of *M. plurianimalium* UK1–20 and *Moraxella* sp. VL6–6 as indicated above. Although enhanced adhesion to epithelial cells was not observed in UK1–20p27 (not shown), this strain showed higher association with PAMs than the original strain (T test, *P* = 0.04; Fig. 6). In contrast, VL6–6p27 showed the same level of interaction with PAMs as the original strain VL6–6 (Fig. 6); this may be due to a different adaptation to laboratory conditions, as observed by the different type of biofilm formed by this strain.

## Discussion

*Moraxella* spp. from the nasal microbiota of piglets showed heterogeneous characteristics, from antimicrobial resistance profiles to virulent mechanisms and adherence properties. The majority of the isolates belonged to *M. plurianimalium* species, but we also detected *M. porci,* a couple of isolates of uncertain classification and a cluster of strains that may constitute a new *Moraxella* species. This cluster comprised strains with a slower growth rate in broth than the rest of the nasal strains and contained most of the strains with high resistance to the serum complement.

Higher rates of antimicrobial resistance were found in strains from farms undergoing antimicrobial treatments, but further studies are needed to demonstrate that the administration of these drugs promotes resistance in this bacterial genus. MDR strains were broadly detected, and only one strain, UK1–20, from a farm where no antibiotics were used, did not show resistance to any of the antimicrobials tested. It is important to highlight the presence of four MDR strains of *Moraxella pluranimalium,* with a high number of drug resistances, including colistin in two of them. This latter observation is consistent with the recent discovery of colistin resistance genes in *Moraxella* [10–12]. Coding genes of antibiotic resistances can be horizontally transferred between bacteria of the same or even different species, and therefore, the presence of resistance genes in the resident microbiota is of concern since they could be transferred to potential pathogens [13]. Diversity of the nasal *Moraxella* isolates was also evident in the in vitro assays, where strains showed a wide heterogeneity.

Biofilms are traditionally considered forms of resistance to environmental conditions and a virulence mechanism in bacteria of clinical importance [14]. As for other characteristics, we observed heterogeneity in biofilm formation capacity of the porcine nasal strains of *Moraxella*. Variability in biofilm formation has been already reported for other species of *Moraxella* from ruminants [15]. Biofilm formation capacity can be associated also to the colonizing ability of the bacteria, as was observed for *H. parasuis* [16]. The interaction of the nasal *Moraxella* strains with the respiratory mucosa needs more study, but attachment to mucin as wells as to epithelial cells could play a role, as suggested by our results.

On the other hand, bacteria from the upper respiratory tract can occasionally reach the lower tract, where they will be confronted with other components of the immune system, such as the alveolar macrophages. In vitro assays with PAMs provided insight into the potential of the nasal isolates to survive this first barrier of cell immunity in the lung. Heterogeneity in this feature has been shown for other bacterial species, such as *H. parasuis* [17] or *Klebsiella pneumoniae* [18]. In our case, strains with high association to PAMs, such as LG6–2, were observed, while other strains did not associate with the phagocytic cells, as the case of the virulent SN9-4 M. According to the results, macrophage association is not directly associated with the phylogeny of the isolates, since heterogeneity in this trait was observed within the distinct species or clusters. One paradigmatic case could be EJ45–1 and SN9-4 M from *M. porci*, having high differences between them. The importance that phagocytosis resistance could have for in vivo infection/colonization by *M. pluranimalium* is supported by the fact that originally the UK1–20 strain showed no association with PAMs (indicative of resistance to phagocytosis), but lost this trait after in vitro culture passages, in agreement with the loss of virulence after laboratory adaptation in other bacteria [19]. Loss of phagocytosis resistance in UK1–20 was concurrent with increase biofilm formation capacity. Thus, biofilm formation may be relevant in environmental persistence, whereas phagocytosis resistance will be important during host infection. However, this phenomenon seems to be strain-dependent, since it was not observed with strain VL6–6, which did not show an adaptation to laboratory conditions that affected its original resistance to PAMs.

Most of the assayed *Moraxella* spp. strains showed sensitivity to the serum complement, with some exceptions, including most of the strains in the cluster representing a putative new species. Thus, most nasal *Moraxella* strains are probably poorly invasive and will be kept on the nasal mucosa, but the new putative species cluster deserves more attention to define their role in disease in pigs.

## Conclusions

The characterization of *Moraxella* spp. isolates from the nasal cavities of young piglets demonstrated a high heterogeneity within this genus. Most of the isolates were identified as *M. pluranimalium*, and only one as *M. porci*. A group of 7 isolates clustered together by 16S rRNA gene sequence and did not show homology to any known *Moraxella* species. Many of the *Moraxella* spp. strains were resistant to multiple antimicrobial classes. Heterogeneity of the *Moraxella* strains was also evident in adhesion and virulence-associated assays. Further analyses, including experimental infections, need to be done to explain the function of this genus in swine health and disease.

## Methods

### Isolation and bacteria cultures

Nasal cavities from 10 piglets of around 3 weeks of age from 5 different litters were sampled from 8 commercial farms (Table 1) with standard management practices for husbandry and welfare of the animals. Sampling of piglets was done under institutional authorization and followed good veterinary practices. According to European (Directive 2010/63/EU of the European Parliament and of the Council of 22 September 2010 on the protection of animals used for scientific purposes) and Spanish (Real Decreto 53/2013) normative, this procedure did not require specific approval by an Ethical Committee. Nasal sampling was performed only once to each piglet and is not likely to cause pain, suffering, distress or lasting harm equivalent to, or higher than, that caused by the introduction of a needle in accordance with good veterinary practice (Chapter I, Article 1, 5 (f) of 2010/63/EU). Nasal swabs were transported in Amies medium to the laboratory, where they were plated on chocolate agar (Biomérieux, Marcy l’Étoile, France) to isolate colonies. After 24 and 48 h of incubation at 37 °C and 5% CO_2_, different colonies were selected and stored at − 80 °C in 20% glycerol-Brain Heart Infusion broth (BHI) for further characterization. Bacterial suspensions were also performed in phosphate-buffered saline (PBS) for DNA extraction.

In addition, reference strains of *Moraxella porci* SN9-4 M and *Moraxella pluranimalium* CD12-CA4*,* both isolated from lesions of diseased pigs, were used in this study. For assays with PAMs, 2 reference strains of *Haemophilus parasuis* were used as controls.

### Bacterial identification and genotyping

DNA extraction was performed using Chelex based Instagene™ Matrix (Bio-Rad Laboratories, Hercules, CA, USA) following manufacturer’s instructions. Preliminary identification of isolates was performed by partial sequencing of the 16S rRNA gene using primers 358F (CTACGGGAGGCAGCAGT) and 907R (CCGTCWATTCMTTTGAGTTT) [20]. Sequences were analyzed by blasting against the Ribosomal database (http://rdp.cme.msu.edu).

All isolates identified as *Moraxella* were then genotyped by ERIC-PCR [21] with primers ERIC-1F (ATGTAAGCTCCTGGGGATTCAC) and ERIC-2R (AAGTAAGTGACTGGGGTGAGCG). PCR reaction mixture consisted of 3 mM of MgCl_2_, 1.2 μM of each primer, 0.23 mM of dNTPs, 0.75 U of GoTaq® polymerase (Promega, Madison Wisconsin, USA) and 100 ng of DNA sample. Amplification was carried out with an initial denaturation of 94 °C for 2 min followed by 30 cycles of 30 s at 94 °C, 1 min at 50 °C and 2.5 min at 72 °C, and a final extension of 20 min at 72 °C. One isolate from each fingerprinting profile was selected for further analysis.

Final identification of the different *Moraxella* strains was performed by 16S rRNA gene amplification with universal primers 8F (AGAGTTTGATCCTGGCTCAG) and 1492R (CGGTTACCTTGTTACGACTT) [22] and sequencing with 8F, 1492R, 358F and 907R primers. Sequence analysis was performed with Fingerprinting II v3.0 software (Bio-Rad) and a UPGMA dendrogram was built for species assignment, including sequences from different *Moraxella* spp. type strains. Biochemical characterization of representative isolates was performed using the Vitek 2 system (Biomerieux).

### Antimicrobial susceptibility

Susceptibility to several antimicrobials was tested as previously described [23]. Neo-Sensitabs™ diffusion tablets (Rosco Diagnostica, Taastrup, Denmark) were used for Gentamicin, Ceftiofur, Colistin, Erythromycin, Lincoespectin, Tetracycline, Doxycycline, Trimetoprim + Sulfamide (T + S), Enrofloxacin, Amoxicillin and Amoxicillin + Clavulanic acid testing. Marbofloxacin, florfenicol and tulathromycin were tested in chocolate agar plates at different dilutions, from 16 to 0.125 μg/mL in the case of florfenicol and marbofloxacin; and from 64 to 0.125 μg/mL in the case of tulathromycin. McFarland suspensions of 0.5 in 0.9% NaCl were prepared for each strain and streaked on the plates with the different antibiotic concentration. Minimal inhibitory concentration (MIC) was determined observing the bacterial growth after 24 h of incubation. As no clinical breakpoints are available for these species, available CLSI breakpoints for *Moraxella catarrhalis* were taken for Tetracycline, Doxycycline, Trimetoprim+Sulfonamide, and veterinary practice according to CLSI breakpoints was followed for the rest of drugs (EUCAST-and CLSI potency NEO-SENSITABS, Rosco Diagnostica, 2013).

### Serum resistance assay

Serum resistance assay was carried out with rabbit serum (EU Directive 2010/63/EU and Spanish normative Real Decreto 53/2013 were followed). A bacterial suspension of each strain (representative of each ERIC fingerprinting) was prepared in PBS with 20% glycerol to reach an OD_600_ of 0.3 in a VIS 7200 spectrophotometer (Dinko Instruments, Barcelona, Spain). In duplicate wells, 10 μl of the bacterial suspension (approx. 10^6^ colony forming units [CFU]/mL) were mixed with 90 ul of fresh filtered rabbit serum and mixtures were incubated for 1 h at 37 °C and 100 rpm. Bacterial survival was calculated by comparing bacterial counts (obtained by serial dilutions and plating) at time 0 and after 1 h incubation. The assay was carried out 3 times for each strain.

### Association to porcine alveolar macrophages

Association to macrophages was tested as previously described with PAMs [17]. PAMs were obtained from healthy piglets euthanized by intravenous pentobarbital overdose under institutional authorization. All procedures involving animals followed EU and Spanish normative (Directive 2010/63/EU and Real Decreto 53/2013). Lungs were removed and PAMs were isolated by lung lavage with PBS containing 70 μg/mL of gentamicin. PAMs were collected by centrifugation at 241 x *g* for 15 min, washed twice with sterile PBS and stored at − 150 °C in 10% Dimethylsulfoxide (DMSO) in fetal bovine serum (FBS) until use.

Selected nasal strains of *Moraxella* were tested to determine their level of association to PAMs. *Moraxella porci* reference strain SN9-4 M was also included in the assay. In addition of two reference strains of *Haemophilus parasuis,* Nagasaki (virulent) and SW-114 (non-virulent), were included as control for the technique.

For the assay, PAMs were plated in Dulbecco’s Modified Eagle Medium (DMEM) supplemented with 10% of FBS and 1% glutamine at a concentration of 5 × 10^5^ cells per well in 6 well-plates. Plates were incubated 1–2 h to allow the attachment of the macrophages to the bottom of the wells. For each strain duplicate wells were inoculated with 10^7^ CFU of fluorescein isothiocyanate (FITC)-labelled bacteria (bacterial inocula were confirmed by dilutions and plating). After 1 h of incubation at 37 °C and 5% CO_2_, plates were transferred to an ice bath to stop interaction and were washed twice with PBS to eliminate unbound bacteria. PAMs were then scrapped in PBS and were analyzed by flow cytometry using an EPICS XL-MCLTM Flow Cytometer (Beckman Coulter, Madrid, Spain) or MACSQuant Analyzer 10 (Myltenyi Biotec, Bergisch Gladbach, Germany). Assays were repeated with PAMs from different animals that were already available at the laboratory. Since each batch of macrophages showed different level of activity, values were harmonized considering the reference *H. parasuis* strain SW114 (phagocytosis sensitive) as 100% phagocytosis in each assay; i.e., using the following calculation: (% PAMs with test bacteria - % fluorescence background in PAMs without bacteria / % PAMs with positive control SW114 - % fluorescence background in PAMs without bacteria) × 100.

### Biofilm assays

Biofilm assays were performed in 96 well cell culture plates under static and shaking conditions following previous published protocol with some modifications (Bello-Ortí et al. 2014). Bacterial suspensions were made to reach an OD_600_ 0.3 in BHI. Wells in 96 well plates were then inoculated in quadruplicate with a 1:100 dilution of the bacterial suspension in BHI. Plates were incubated at 37 °C, under static conditions with 5% CO_2_ or under agitation at 100 rpm for 24 and 48 h, except for slow growing isolates, which were incubated for 48 and 72 h. After incubation, wells were emptied and rinsed with tap water to remove unattached bacteria. Wells were then stained with 0.1% (w/v) crystal violet (Merck, Darmstadt, Germany) for 2 min at room temperature. Wells were washed thrice with tap water to remove the excess of crystal violet and let dried at 37 °C. The dye in the stained biofilms was solubilized with 100 μL of 70% ethanol and quantified at 590 nm in a Powerwave XS Microplate Spectrophotometer (Biotek Instruments Inc., Winooski, VT, USA). Biofilm formation was also assessed on wells pre-coated with 5 μg of mucin or BSA as control.

### Cell adhesion assay

Porcine epithelial cells PK-15 (ATCC® CCL-33) and human lung A549 (ATCC® CCL-185) cell lines were cultured in DMEM supplemented with 1% glutamine and 5% FBS for PK-15 and 10% for A549 cells.

Concentration of cells per ml was calculated to obtain a monolayer after overnight incubation, approximately 50,000 cells/well in cell culture 96 well plates. After overnight incubation, wells with PK-15 or A549 cells were washed once with sterile PBS. Duplicate wells were inoculated with 10^7^ CFU/mL bacteria from each isolate tested. Bacterial inoculum quantification was confirmed by colony counts. Microplates were then centrifuged 10 min at 100 x *g* to facilitate the contact between bacteria and cells. After incubation for 1 h at 37 °C and 5% CO_2_, wells were washed twice with sterile PBS to remove unattached bacteria, and attached bacteria were released with 0.1% of saponin and pipetting. The resulting suspension was quantified by plating dilutions on agar plates.

## Supplementary information

**Additional file 1.** Biochemical characterization by Vitek 2 (Biomerieux) of representative strains.

## Data Availability

The datasets analysed during the current study are available from the corresponding author on reasonable request.

## Abbreviations

BHI: Brain Heart Infusion
BSA: Bovine serum albumin
CFU: Colony forming units
DMEM: Dulbecco’s Modified Eagle Medium
DMSO: Dimethylsulfoxide
ERIC: Enterobacterial repetitive intergenic consensus
FBS: Fetal bovine serum
FITC: Fluorescein isothiocyanate
MDR: Multidrug-resistant
MIC: Minimal inhibitory concentration
PAMs: Porcine alveolar macrophages
UPGMA: Unweighted pair group method with arithmetic mean
