# Supplementary material for: Heterogeneity of Moraxella isolates found in the nasal cavities of piglets
Source: BMC Vet Res. 2020 Jan 30;16:28. doi: 10.1186/s12917-020-2250-9 (PMC6993494; doi:10.1186/s12917-020-2250-9)
Supplement: Supplementary file 1 — Additional file 1. Biochemical characterization by Vitek 2 (Biomerieux) of representative strains. [file 12917_2020_2250_MOESM1_ESM.docx]

**Additional file 1**. Biochemical characterization by Vitek 2 (Biomerieux) of representative strains.

|  | *Moraxella pluranimalium* | | *Moraxella porci* | |  |  |
| --- | --- | --- | --- | --- | --- | --- |
| **Test** | CD12-CA4 | LG6-2 | SN9-4M | EJ45-1 | CR-7A | VL1-5 |
| Ala-Phe-Pro-Arylamidase | - | - | - | + | - | - |
| Adonitol | - | - | - | - | - | - |
| L-Pyrrolydonyl-Arylamidase | - | - | - | - | - | - |
| L-Arabitol | - | - | - | - | - | - |
| D-Cellobiose | - | - | - | - | - | - |
| Beta-Galactosidase | - | - | - | - | - | - |
| H_2_S Production | - | - | - | - | - | - |
| Beta-N-Acetyl-Glucosaminidase | - | - | - | - | - | - |
| Glutamyl Arylamidase pNA | + | + | - | + | + | + |
| D-Glucose | - | - | - | - | - | - |
| Gamma-Glutamyl-Transferase | - | - | - | - | (-) | - |
| Fermentation / Glucose | - | - | - | - | - | - |
| Beta-Glucosidase | - | - | - | - | - | - |
| D-Maltose | - | - | - | - | - | - |
| D-Mannitol | - | - | - | - | - | - |
| D-Mannose | - | - | - | - | - | - |
| Beta-Xylosidase | - | - | - | - | - | - |
| Beta-Alanine Arylamidase pNA | - | - | - | - | - | - |
| L-Proline Arylamidase | - | - | - | + | - | (-) |
| Lipase | - | - | - | + | - | - |
| Palatinose | - | - | - | - | - | - |
| Tyrosine Arylamidase | - | + | + | + | - | + |
| Urease | - | - | - | - | - | - |
| D- Sorbitol | - | - | - | - | - | - |
| Saccharose / Sucrose | - | - | - | - | - | - |
| D-Tagatose | - | - | - | - | - | - |
| D- Trehalose | - | - | - | - | - | - |
| Citrate (sodium) | - | - | - | - | - | - |
| Malonate | - | - | - | - | - | - |
| 5-KetoD-Gluconate | - | - | - | - | - | - |
| L-Lactate alkalinisation | - | - | - | - | - | - |
| Alpha-Glucosidase | - | - | - | - | - | - |
| Succinate alkalinisation | - | - | - | - | - | - |
| Beta-N-Acetyl-Galactosaminidase | - | - | - | - | - | - |
| Alpha-Galactosidase | - | - | - | - | - | - |
| Phosphatase | - | - | - | - | - | - |
| Glycine Arylamidase | - | - | - | + | - | - |
| Ornithine Decarboxylase | - | - | - | - | - | - |
| Lysine Decarboxylase | - | - | - | - | - | - |
| Decarboxylase base | - | - | - | - | - | - |
| L-Histidine assimilation | - | - | - | - | - | - |
| Coumarate | - | - | - | - | - | - |
| Beta-Glucoronidase | - | - | - | - | - | - |
| O/129 Resistance (comp.vibrio.) | - | - | - | - | - | - |
| Glu-Gly-Arg-Arylamidase | - | - | - | - | - | + |
| L-Malate assimilation | - | - | - | - | - | - |
| Ellman | - | - | - | + | - | - |
| L-Lactate assimilation | - | - | - | - | - | - |
